# How much does it cost to prevent and control visceral leishmaniasis in Brazil? Comparing different measures in dogs

**DOI:** 10.1371/journal.pone.0236127

**Published:** 2020-07-21

**Authors:** Anaiá da Paixão Sevá, Fernando Ferreira, Marcos Amaku

**Affiliations:** 1 Department of Animal Health and Preventive Veterinary, University of São Paulo, São Paulo, São Paulo, Brazil; 2 Department of Exact and Technological Sciences, State University of Santa Cruz, Ilhéus, Brazil; University of Lincoln, UNITED KINGDOM

## Abstract

Visceral leishmaniasis (VL) is an important zoonosis in Brazil. Dogs are considered the main domestic reservoirs of the disease in the country; hence, control measures are focused on these reservoirs. Despite efforts to prevent and control VL, important reductions in disease prevalence and incidence have not been identified, stimulating the development and application of new strategies. The choice and implementation of new control strategies can benefit from the application of mathematical models that allow the simulation of different strategies in different scenarios. Selecting the best strategy to be implemented is also supported by cost-effectiveness studies. Here we used the results of a mathematical model in which scenarios, including isolated use of the vaccine and insecticide-impregnated collar (IIC), both at different coverage rates, were simulated to conduct a cost-effectiveness study. The costs were calculated for each scenario considering a simulation period of four years. Collar application in both infected and non-infected animals was the most cost-effective strategy. For example, to reduce the prevalence in humans and dogs by approximately 70%, the costs ranged from $250,000 and $550,000 for the IICs and vaccination, respectively. Even in the scenario with 40% loss/replacement of IICs, this measure was more advantageous in terms of cost-effectiveness than vaccination. If the vaccine were applied with culling of seropositive tested dogs, then the measure became more effective with a reduced cost compared with the vaccine alone. The use of the three first consecutive vaccine doses had the greatest impact on the cost of the vaccination strategy. The advantage of using IICs is that there is no need for a prior diagnosis, unlike vaccination, reducing costs and facilitating implementation. The present study aims to contribute to strategies to reduce hosts infected with VL by reducing public expenditure.

## Introduction

Visceral leishmaniasis (VL) is an important zoonosis in both humans and domestic dogs in Brazil [1]. Approximately 80% of human VL cases worldwide are reported in Brazil [2], impacting society and the public health system [3]. Although the Brazilian Visceral Leishmaniasis Surveillance and Control Program (VLSCP) has existed for more than ten years, the area of occurrence of the disease is expanding geographically, and there have been no notable reductions in disease incidence [4–6].

Domestic dogs are considered the main reservoir of VL in Brazil and have an important role in disease epidemiology [7]. Additionally, the high prevalence of infection in dogs is associated with an increased risk of human cases [7]. Since 2002, one measure recommended by the VLSCP is euthanasia of seropositive animals; the other two are the diagnosis and immediate treatment of humans and the use of residual insecticides to control the vector [1].

Although few studies have reported positive results of euthanasia and its great theoretical effectiveness, when applied properly [8], this measure is questionable due to its efficacy, applicability, and insalubrity as well as ethical reasons, which involve animal welfare and the health of the people who perform and are involved in this task [9–15]. There is also resistance in the population, including animal protectors, activists, and owners, who do not want to euthanize the animals, especially when they are infected but clinically healthy [16]. This resistance is aggravated by the occurrence of diagnostic failures due to limitations in the sensitivity and specificity of diagnostic tests used before euthanasia [17]. All these issues make makes difficult to implement euthanasia by the VLSCP in several areas of Brazil [16]. The application of this measure for almost ten years would allow one to discern whether the execution of capacity has been applied in its maximum effort, with the prevalence of the disease in its hosts still showing no significant reduction. Consequently, new strategies for prevention and control have been evaluated, and previous strategies have been reanalyzed [18].

Field studies have demonstrated the effectiveness of insecticide-impregnated collars (IICs) in seronegative dogs [16,19–22] and vaccines in dogs [20,23–25], as well as theoretical studies [25–27]. However, only one study has evaluated the costs of the strategies, but without considering previous serodiagnostic assessments of the three initial doses and the mortality of the vector caused by collar [28].

In the present study, the costs of these two strategies were evaluated for dogs (IIC and vaccine) in which the VLSCP had not yet been implemented, as suggested new options that are considered ethics and feasible for implementation. Thus, the impact on prevalence reduction in the involved hosts (humans and dogs) and operational and technical aspects were evaluated. To perform the evaluations, the results of a mathematical model developed to evaluate the effectivity of these measures were analyzed [25].

## Methodology

Using a mathematical model applied to VL in Brazil, we simulated interventions not used by VLSCP until now, such as vaccination and IIC use, in the dog population at different coverage rates (20%, 40%, 60%, 80%, and 95%). The costs of these measures were calculated for a four-year simulation.

### Model and biological parameters

The mathematical model developed by Sevá et al. [25] was fitted to a community with 45,000 humans and 3,000 dogs (dogs equivalent to 15% of humans [29]) and an endemic scenario in Brazil similar to a symptomatic human cases rate of 0.046% and seropositivity rate of 27% in dogs. The premises of the previous model, including constant populations, equal probability of infection for each host, and absence of seasonality, were maintained in this model.

Since vaccination was performed based on the serological status of the dogs, we included the cost of serological tests in the cost of this strategy. In addition, according to the vaccine manufacturer's protocol, primary vaccinated dogs received three doses in the first year (with 21-day intervals between each vaccination), followed by annual doses [30]. Thus, the model was composed of one compartment for primary vaccinated animals and three additional compartments for animals with annual revaccination, totaling four compartments, one for each year. The model is presented in S1 Fig, with its data at Table 1 and the full description is available in the supplementary file (S1 File). The current strategy proposed by the government consists of euthanasia of seropositive dogs; however, the average coverage of euthanized individuals is 60% [8]. The vaccination strategy requires prior testing of the dogs. Therefore, the model simulations were performed without and with 60% euthanasia of seropositive (really and false positives) tested animals with previous vaccination.

**Table 1:** Coverage of each measure, and prevalence reductions at the end of four years compared with the initial prevalence (0.046% of symptomatic humans and 27% of seropositive dogs, without control measures) and their associated costs.

Measure	Dog coverage (number of animals)	Dog coverage of euthanasia (number of animals)	Sick humans		Seropositive dogs		Costs of	Costs with high collar
			(%)	Nº	(%)	Nº	measure ($)	lost ($)
None			0.046	21	26.7	801		
COL	2,469		0.035	16	19.8	594	$53,739	$74,165
	5,135		0.028	13	14.8	444	$109,983	$152,460
	8,153		0.021	9	10.3	309	$173,564	$241,008
	11,962		0.014	6	6.2	186	$253,611	$352,568
	16,736		0.008	4	3.2	96	$353,717	$492,161
VAC	1,660		0.035	16	16.6	498	$173,621	
	2,154		0.032	14	10.7	321	$225,246	
	3,317		0.024	11	7.1	213	$402,971	
	5,151		0.018	8	4.8	144	$537,032	
	6,080		0.011	5	3.7	111	$634,212	
VAC + EUT	1,732	313	0.027	12	13.8	414	$173,946	
	2,228	244	0.022	10	10.6	318	$219,357	
	4,088	378	0.009	4	2.9	87	$396,536	
	5,530	450	0.003	1	0.6	18	$532,344	
	6,097	473	0.002	1	0.2	6	$581,258	

IIC: Insecticide-impregnated collar; VAC: vaccine; $: American currency (Dollar).

### Cost calculation

For vaccination, diagnostic tests, such as the rapid DPP^®^ test for detection and ELISA^®^ for confirmation, as recommended by VLSCP, were included for the primary vaccinated dogs. De Carvalho et al [31] evaluated both tests in an area with a canine seroprevalence of 30%, similar to that in the present study, and identified that of the 140 dogs, 42.8% (60) were positive using the detection test, of which 83.3% (50) were positive using the confirmatory test. Thus, we calculated that vaccinating 100 animals is necessary to test 156 animals with both diagnostic tests. The collars were considered to be applied twice a year, according to the manufacturer's protocol, for all dogs (seronegative and seropositive).

For the vaccination and application of the IICs, we considered the implementation of fixed service points with the presence of veterinarians and support technicians from the city halls distributed throughout the municipality. At these stations, animals and guardians are registered, and individuals receive a certificate after applying the product to the dogs.

The costs for replacing 40% of the collars resulting from loss and damage were also simulated.

The calculation of product and trained personnel costs and currency conversion were based on exchange rates in April 2019 (S2 File).

## Results and discussion

### Collar

Two annual applications of the collar were considered since the product lasts for six months. Thus, at the end of four years, 2,470, 5,136, 8,158, and 11,969 collars were applied, corresponding to coverage rates of 20%, 40%, 60%, and 80%, respectively.

The results of simulating the use of collars demonstrated considerable reductions of human cases and seropositive dogs (Table 1). Field studies in Brazil also revealed an effective reduction of the seroprevalence of dogs when applying this strategy. Two types of IICs with different active products, which are currently available on the market, and effectiveness rates after one year of follow-up were 88.3% and 61.8% in the Seresto^®^ (10% imidacloprid/4.5% flumethrin) and Scalibor^®^ (4% deltamethrin) groups, respectively [19]. Additionally, other studies used IICs (4% deltamethrin) in dogs and observed a measure effectiveness of 63% [16], 57.7% [20], and 44.7% [32]. Maroli et al. observed an effectiveness rate of 50% after the 1^st^ year and 86% after the 2^nd^ year of application [21]. Coura-Vital et al. also found that the effectiveness of the strategy increased with an increased intervention period [16]. As found in the present study, the number of human cases and seropositive dogs decreased gradually over time.

Unlike the cited field studies, we simulated IIC use in all dogs in the population, independent of their clinical-immunological status and without concomitant measures, such as euthanasia of seropositive individuals. Thus, even when keeping infected animals in the population, the strategy produced a significant reduction of human cases and seropositive dogs.

The costs of collaring with lost/replaced IICs was considerable at approximately 39% higher than without lost/replaced IICs (Table 1), suggesting that it is necessary to manage the survey of these losses as well as to immediately replace lost collars. We also recognize that this action represents an operational complexity of this strategy. In the study by Leite et al., it was not considered possible to immediately replace the collar due to logistic difficulties and the high cost of the product [32]. In previous studies on the use of IICs in domestic dogs, the rate of collar loss varied from 4.9% to 64.7% [16,32]. Researchers attributed these collar losses to different reasons: 1) removal by the owners due to allergies or dermatitis; 2) fights between animals; 3) removal by the dogs themselves, especially semi-domestics who were not used to wearing collars; and 4) removal by the owners for resale. In the field study by Coura-Vital et al., it was concluded that to maintain the effectiveness of the collar, strategies must be adopted that allow the rapid replacement of lost IICs [16]. Thus, we also calculated the cost-effectiveness of the measure considering an IIC loss rate of 40%. However, regarding the cost of replaced collars, our simulations demonstrated a cost-effective advantage when compared with the vaccination strategy. Conversely, we must highlight that the IIC strategy needs to be associated with education focused on the importance of the maintenance of collars on dogs and its relevance for disease prevention in humans.

We proposed the use of fixed station points to reduce the costs associated with displacement of work teams and their return to properties that are momentarily empty. In previous field studies, the IICs were applied to dogs in their domiciles [16,20,32,33]. According to Alves et al., the domiciles were empty in 22.6% of the visits, characterizing an operational difficulty [33]. However, the disadvantage of our proposal is the dependence on the owner to actively obtain the product, which can impact the number of collars applied.

### Vaccine

Simulating the application of the vaccine at different coverage rates, it was observed that the number of animals that received all three doses in the first year of vaccination was much higher than those that received an annual revaccination (Table 2). In a population with low life expectancy, a high turnover of animals and a large number of puppies are common [34]. Thus, we considered that to reduce the measure costs, it could be necessary reduce the number of new individuals in a population, improving their quality of life through responsible owner education, increasing their life expectancy, and even reducing the birth rate.

**Table 2:** Vaccinated dogs according to year and applied doses in a population of 3,000 animals.

	Number of vaccinated dogs		Total of doses
Coverage (%)	Initial doses	Revaccination	
20	1,695	534	5,619
40	3,089	998	10,265
60	4,161	1,368	13,851
80	4,582	1,515	15,261

In addition to the high cost of vaccination resulting from the large number of doses applied to primary vaccinated dogs, the high rate of animal replacement translates into operational complications for completion of the initial vaccination schedule, which involves an initial serodiagnostic test and requires confirmation that the dog owner takes him to the service station for 3 consecutive doses at 21-day intervals.

In the present study, vaccination at different coverage rates significantly reduced human cases and seropositive dogs (Table 1). However, vaccination has not been used as a measure to control VL due to the lack of evidence regarding the vaccine’s effectiveness [35]. In field studies, vaccine effectiveness varied from 32.8% [20] to 71.4% [23]. Fernandes et al. found a higher effectiveness of 85.2%, but this value was determined by evaluating the infectivity of the dog and not the infection rates [24]. In addition, in areas with a high rate of infection, dogs have a high risk of becoming infected before completing the initial three-dose course and developing immunity.

In the present study, dogs with false-negative results were vaccinated because the sensitivity of the test is less than 100%. Although these doses were "lost" in terms of not promoting protection to these already infected animals, the number of doses was low, accounting for nearly 10% of those vaccinated at the end of 4 years.

Considering the application of euthanasia of seropositive animals together with vaccination, the measure was significantly better in terms of cost and effectiveness, even with the low absolute number of euthanized animals (Table 1). However, we must consider that the purpose of applying the vaccine and collar is to eliminate the strategy of euthanasia at the VLSCP, because it is highly questionable for ethical reasons and due to implementation difficulties. In addition, it is known that euthanasia coverage is low in some areas of the country [8].

### Comparison of measures

**Fig 1:**
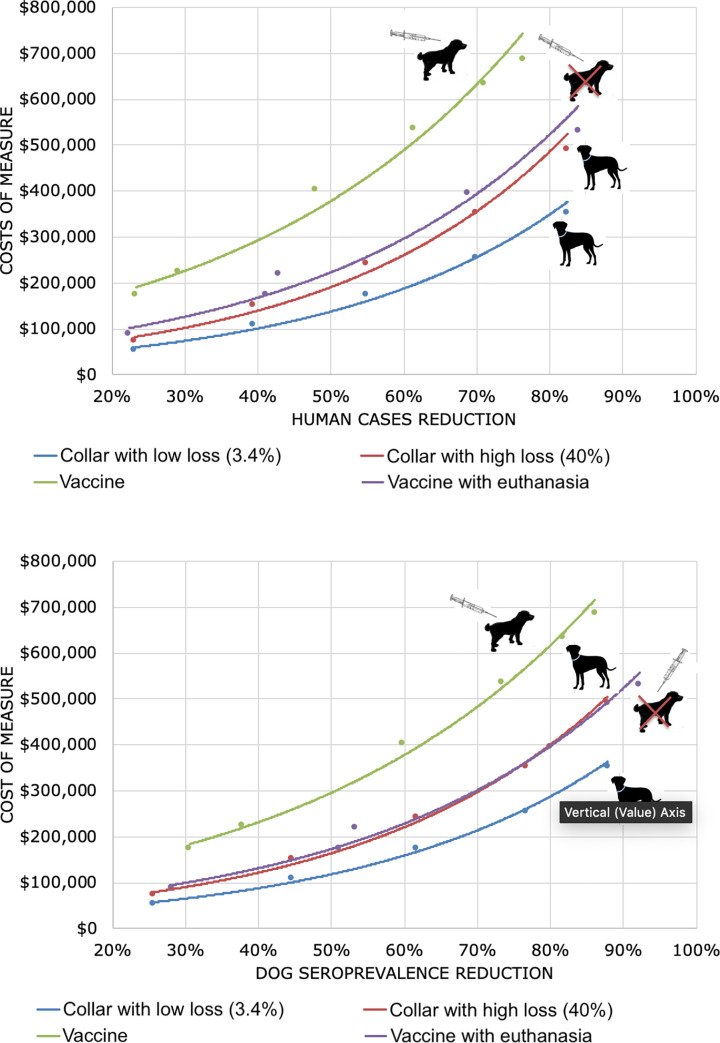
Reductions of the prevalence in dogs (A) and humans (B) in relation to the initial prevalence (0.046% of symptomatic humans and 27% of seropositive dogs, without control strategies) and the respective costs according to each prevention and control strategy. $: American currency (Dollar); “Collar with loss or damage” means that 40% of insecticide-impregnated collars were lost or damaged.

For IICs and vaccination, the reductions of seropositive dogs and human cases were similar; however, the cost of collar (with low and high costs) use was lower than the cost of vaccination (Fig 1).

A complicating factor in the use of the vaccine is the need for prior diagnostic testing. It should be noted that confirmatory tests are usually performed in laboratories far from municipalities in endemic areas of Brazil [14,24]. This long distance results in an increase in the time needed for diagnosis, which can reach up to 60 days [36], a period in which a negative animal can become infected, decreasing the effectiveness of the strategy. In this sense, use of the CII is operationally simpler and more effective than vaccination since there is no need for a prior diagnosis and the intervention has an almost immediate effect.

We also suggest the sterilization of dogs as a measure concomitant with vaccination as a complementary action to reduce the number of stray and new dogs. We can consider that the castration of domiciled dogs also contributes to the reduction of animal abandonment and, therefore, reduces the number of stray dogs. Conversely, this action can also be useful for the IIC strategy, as suggested by Coura-Vital et al., since the use of only IICs in domiciled dogs is not sufficient to control VL because stray dogs are important reservoirs in the environment [16]. However, the fraction of animals without any supervision tends to be low in the dog population, as a high percentage of the dogs can be considered as community dogs and dogs of homeless people. Thus, our simulations did not achieve 100% coverage because it is impossible, mostly due to the presence of stray dogs; however, with a strong effort to achieve coverage, both measures are able to reduce the host prevalence at almost 100%.

### Other considerations

Currently, other VL prevention products are focused on dogs available on the market, such as *pour-on* ampoules with insecticidal and repellent action, similar to the collar. However, their active period is short (approximately one month) compared with IICs (approximately six months). Thus, we chose not to simulate this measure due to the operational difficulty of its implementation as a public policy.

A comparison of the cost-effectiveness of measures not yet used with already existing measure, such as euthanasia, would be possible if it was implemented properly and if the data for implementation were computed correctly. Thus, with the current data of euthanasia, it becomes difficult to calculate the effectiveness in terms of cost, as well the real coverage of animals, the level of interference prolonging the maintenance time of infected remains in the environment, and the prevalence before and after the measure. A comparison with euthanasia would be effective as a proposal if euthanasia were being applied consistently with the theory, in a continuous manner, and without the diagnostic and management problems, as proposed in a previous study (Sevá, et al., 2017).

Since the prevention strategies simulated in the present study reduced the number of human symptomatic cases, denoting those who need treatment, public health spending was thus reduced. In a study conducted in 2014, the cost of VL humans cases in Brazil, including diagnostic and treatments, corresponded to US$ 1,873,682 on average (1,872,131 to 1,882,921), with the majority of expenses associated with hospitalization (40%), followed by treatment (22%) and prophylaxis (18%) [3]. The authors also found a mean cost for diagnostic, VL treatment and hospital and ambulatory care for a VL case of US$ 500.91 (499.95 to 504.57).

Since we are proposing public policy actions, we consider the expenditures with inputs (medicine materials, diagnostic kits, vaccine, and collar) to be the same for the whole country, since funding is provided by the ministry of health. However, variations in measure costs depend on the average salary of the veterinarian and other professionals, which varies in each municipality of the country. Although a base medium salary of U$1,118 was considered for comparative measures, the median minimum and maximum salary are U$260 and U$1,118, respectively [37,38]. However, these different ranges of total costs do not affect comparisons between measures, as both of them depend on these professionals and their salary is the same at the municipality level independently of strategy.

We must highlight that in the present model we did not consider wild hosts at the VL dynamic like the model proposed by Seva et al. [39]. In fact, wild mammals are also a reservoir of the VL agent in Brazil [40], but the both epidemiological role and real prevalence of them have been not clear in this country [41]. In the mathematical model, their prevalence directly affects the prevalence of the vector, which in turn will affect the measure's coverage effort to reduce human and dog VL cases. However, increasing or decreasing the coverage of the strategy will not affect the comparison of the measure's effectiveness, since increasing the coverage of one measure will require increasing the other, and the costs of both would increase accordingly. Thus, the influence of infected reservoirs will be the same for each measure.

As observed in this study and in a previous study on mathematical models applied to the prevention and control of VL [25], it is important that control measures be applied with high coverage and on a continuous basis to ensure effectivity.

## Conclusion

In the present study, the most cost-effective method for controlling VL was the use of IICs, followed by vaccination. It is worth mentioning that in addition to the cost-effectiveness of the strategies used to control VL, a relevant aspect for choosing the strategy to be adopted is the ease of its implementation from operational and technical perspectives.

## Supporting information

S1 Fig Model framework of compartments and flow between them regarding the disease dynamics and the prevention and control measure influences. blue: human populations; pink: vector populations; yellow: dog populations without interventions (control and prevention measures); red: vaccinated dogs; green: dogs with collars.
(TIFF)

S1 File (DOCX)

S2 File (DOCX)
