# Supplementary figures and images for: How much does it cost to prevent and control visceral leishmaniasis in Brazil? Comparing different measures in dogs
Source: PLoS One. 2020 Jul 21;15(7):e0236127. doi: 10.1371/journal.pone.0236127 (PMC7373293; doi:10.1371/journal.pone.0236127)

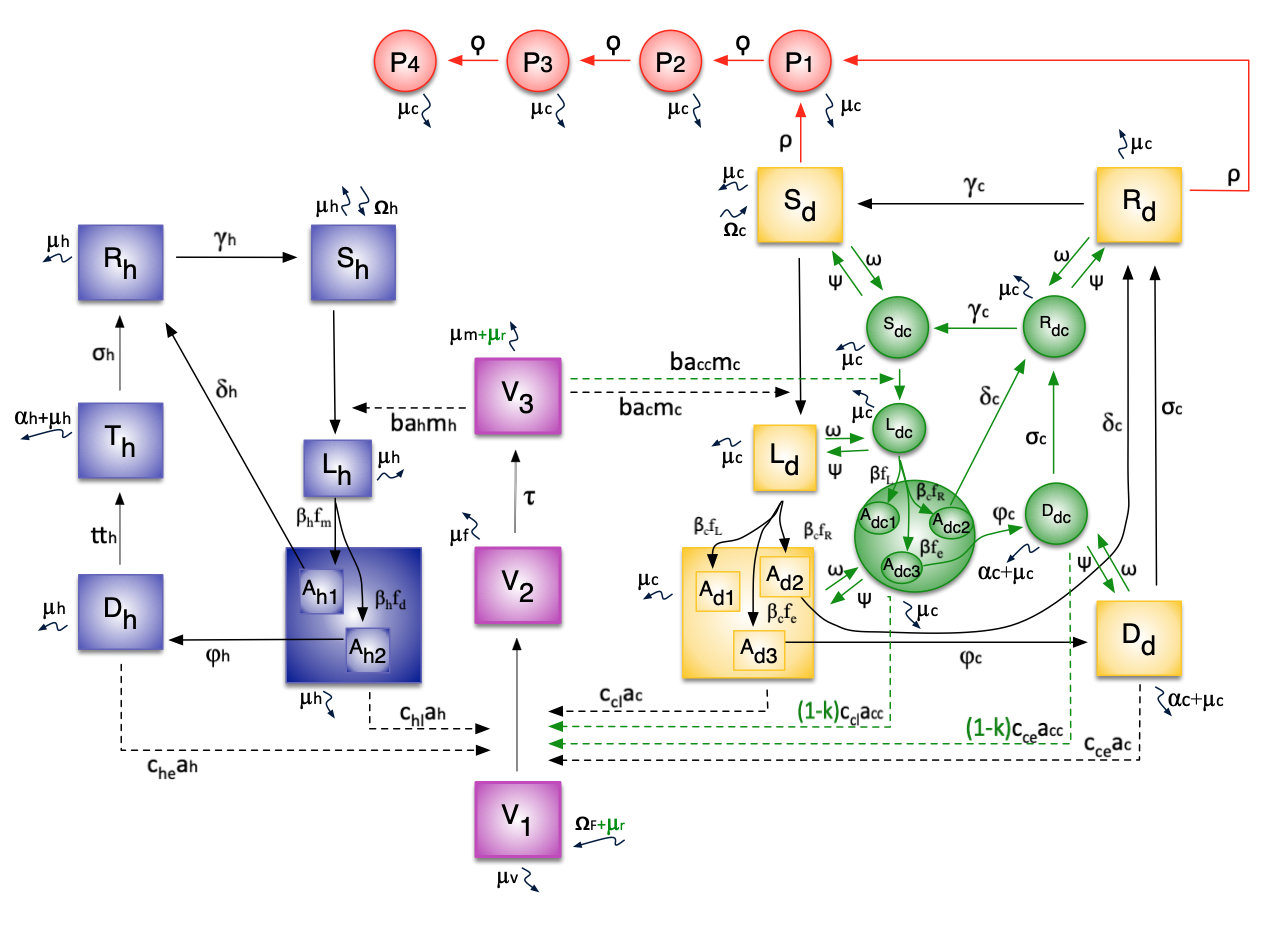

Supplement: S1 Fig — blue: human populations; pink: vector populations; yellow: dog populations without interventions (control and prevention measures); red: vaccinated dogs; green: dogs with collars. (TIFF) [file pone.0236127.s001.tiff]
