# Supplementary material for: How much does it cost to prevent and control visceral leishmaniasis in Brazil? Comparing different measures in dogs
Source: PLoS One. 2020 Jul 21;15(7):e0236127. doi: 10.1371/journal.pone.0236127 (PMC7373293; doi:10.1371/journal.pone.0236127)
Supplement: S1 File — (DOCX) [file pone.0236127.s002.docx]

**Table 1 – Hosts in their various stages, with their clinical and immunological characteristics, and including control measures, and vectors in their respective stages with the parasite**

| **HUMANS** | | |  | |
| --- | --- | --- | --- | --- |
| **S_h_** | **Susceptible:** | | | **Not infected.** |
| **L_h_** | **Latent:** | | | **Infected and non infectious. Asymptomatic and with negative immunity responses.** |
| **A_h1_ e A_h2_** | **Asymptomatic:** | | | **Infected and infectious. Asymptomatic and with positive humoral and negative cellular immunity responses.** |
| **D_h_** | **Sick:** | | | **Infected and infectious. Symptomatic and with positive humoral and variable cellular immunity responses.** |
| **T_h_** | **Treated:** | | | **Infected and non infectious. On treatment until considerable loss of humoral immunity responses.** |
| **R_h_** | **Recovered:** | | | **Infected and non infectious. Asymptomatic and with negative humoral and positive cellular immunity responses.** |
| **CÃES** | |  | | |
| **S_c_** | **Susceptible:** | | | **Non infected.** |
| **L_c_** | **Latent:** | | | **Infected and non infectious. Asymptomatic and with negative immunity responses.** |
| **A_c1_, A_c2_ e A_c3_** | **Asymptomatic:** | | | **Infected and infectious. Asymptomatic and with positive humoral and negative cellular immunity responses.** |
| **D_c_** | **Sick:** | | | **Infected and infectious. Symptomatic and with positive humoral and variable cellular immunity responses.** |
| **R_c_** | **Recovered:** | | | **Infected and non infectious. Asymptomatic and with negative humoral and positive cellular immunity responses.** |
| **DOGS WITH INSECTICIDE IMPREGNATED COLLAR** | | | | |
| **S_cc_** | **Susceptible:** | | | **Susceptible with collar** |
| **L_c_** | **Latent:** | | | **Latent with collar** |
| **A_cc1_, A_cc2_ e A_cc3_** | **Asymptomatic:** | | | **Asymptomatic with collar** |
| **D_cc_** | **Sick:** | | | **Sick with collar** |
| **R_cc_** | **Recovered:** | | | **Recovered with collar** |
| **DOGS PROTECTED WITH VACCINE** | | | | |
| **P1** | **Vaccinated at the first time** | | | |
| **P2, P3, P4** | **Annual revaccination** | | | |
| **VECTORS** | | |  | |
| **V_1_** | **Non infected.** | | | |
| **V_2_** | **Infected but not infective** | | | |
| **V_3_** | **Infected and infective** | | | |

**MODEL DESCRIPTION**

**Humans**

**Humans are born susceptible (S_h_) at a rate of** Ω_h_ **and are infected at a rate of** ba_h_m_h_V_3_, in which b is the proportion of infective bites, a_h_ is the average number of daily vector bites on humans, and m_h_ is the vector density per human host (equations 1 and 2). Following infection, humans become infected but are not infective; thus, they enter compartment L_h_. Fraction f_m_ of the infected, non-infective individuals remains asymptomatic at the compartment A_h1_ until they recover from the disease, and move to that a rate of β_h_f_m_, whereas fraction f_d_ develops symptoms and moves to compartment A_h2_ at a rate of β_h_f_d_ (equations 2-4). The individuals in compartments A_h1_ and A_h2_ are considered to be asymptomatic, and they are discriminated according to their next stage. The value of β_h_ is given by the inverse of the time an individual remains non-infective and lacks humoral immunity. The infective individuals in compartment A_h1_ become resistant as a function of the development of cell-mediated immunity, and they move to compartment R_h_ at a rate of δ_h_, whereas the infective individuals in A_h2_ develop disease at a rate of φ_h_ and move to the compartment of the diseased individuals, D_h_. It was assumed that the diseased individuals are hospitalized and thus move to compartment T_h_ at a rate of tt_h_, losing their infective capacity. At the hospital, humans may either die from VL at a rate of α_h_ or recover, whereby they move to compartment R_h_ at a rate of σ_h_. With the loss of cellular immunity, the recovered individuals become susceptible again, thus moving to compartment S_h_ at a rate of γ_h_. All humans are subjected to the natural mortality rate μ_h_ (equations 1-7).

**Dogs**

**Dogs are born susceptible, S_d_, at a rate of** Ω_d_ **and enter compartment L_d_ when they are infected, which occurs at a rate of** ba_c_m_c_V_3_, in which b is the proportion of infecting bites, a_c_ is the average number of daily vector bites on dogs, and m_c_ is the vector density per canine host (equations 8 and 9). Following infection, dogs are infected but not infective in this compartment; a fraction (f*_l_*) of these individuals remain asymptomatic for their entire lives and move to compartment A_d1_, whereas a fraction (f_r_) remain asymptomatic at the compartment A_d2_ until they recover from the disease. Those that develop symptoms (the fraction f_e_) move to compartment A_d3_. The individuals in A_d1_, A_d2_ and A_d3_ are considered asymptomatic, and they are discriminated according to their next stage. The development of infectiveness and humoral immunity, with the move of the individuals in compartment L_d_ to compartments A_d1_, A_d2_ and A_d3_, occurs at rates of β_c_f_l_, β_c_f_r_ and β_c_f_e_, respectively (equations 9-12); the value of β_c_ is given by the inverse of the time an individual remains non-infective and lacking humoral immunity. Individuals A_d2_ become resistant as a function of the development of cell-mediated immunity, and they move to compartment R_d_ at a rate of δ_c_, whereas individuals A_d3_ become diseased at a rate of φ_c,_ thus moving to compartment D_d_. These latter individuals may die from the disease at a rate of α_c_ or recover (R_d_) at a rate of σ_d_. The recovered individuals lose their cellular immunity and become susceptible **(S_d_)** at a rate of γ_d_ (equation 13). The treatment of diseased animals was not considered. All dogs were subjected to the natural mortality rate μ_c_ (equations 8-14).

**The prevention and control methods are described next.**

**Insecticide-impregnated Collar**

**The use of collars as a preventive and control measure was simulated by assuming a rate of application** ω to all of the dog population compartments, which is applied independent from the clinical-immune status of the animals. The collars have two effects: 1) they inhibit vector bites, and 2) they cause the death of the insects that bite the animals wearing them [23]**. The effect of bite inhibition manifests as a reduction of the value of a_c_, which becomes a_cc_ (equations, 15, 16, 23, and** 24). The mortality resulting from the collar insecticide effect was considered instantaneous; k represents the proportion of insects that die after biting a collar-wearing dog. As a result, the rate of infection of insects that bite collar-wearing dogs in compartments A_dc1_, A_dc2_ and A_dc3_ **is represented by c_cl_a_cc_(1-k), whereas the rate corresponding to the insects that bite collar-wearing dogs in compartment** D_dc_ **is represented by c_ce_a_cc_(1-k) (see equations 23 and 24).**

After feeding on collar-wearing dogs and eventually infecting them, vectors (V_3_) die at a rate of µ_r_ as a result of the collar-induced mortality (equation 25). The rate of the loss of efficacy of the collars is represented by ψ (equations 15-21).

Data regarding both repellent and vector mortality effects were considered related to studies of collar impregnated with 4% of deltamethrin (DAVID et al., 2001).

Vaccine

According to the manufacturer's recommendations the vaccinated animals need to be seronegatives (susceptible and recovered). When these animals are vaccinated they go to protected compartment (P_1_), at a rate ρ. This rate takes into account the animal coverage, vaccine efficacy and the specificity (E) of diagnostic tests recommended by the Brazilian Program for Control Visceral Leishmaniasis (BPCVL), such as the trial test DPP® and the confirmatory ELISA®. However, these diagnostic tests also identify false negative individuals, which are considered just for the cost calculations, because they will not be protected, once they are already infected. In that case, the infected dogs (Ac, Lc e Dc) are being vaccinated at a rate ζ, with consider the measure coverage and 1-S (where S is the sensitivity of tests used sequentially).

According to the manufacturer's recommendations, all vaccinated dogs need to be revaccinated annually, so animals in P_1_ go to compartments P_2_, P_3_ and P_4_, representing each consecutive year, at a rate ϙ (inverse of one year period). In all vaccinated compartments, dogs die at a rate µ_c_, of natural mortality (equations 25-28).

Dogs in P_1_ need to receive three doses, according to manufacturer's recommendations. So to cost calculations the total of vaccinated animals in the P_1_ compartment are multiplied for three. The total doses of revaccinations are the sum of P_2_, P_3_ and P_4_ compartments.

Euthanasia

The euthanasia is performed in seropositive animals according to the recommendation of BPCVL. So the asymptomatics and sick dogs are euthanized in a rate μ_s_, in what is also included the sensitivity (S) of diagnostic tests (DPP® and ELISA®) used sequentially as recommended by the BPCVL . However, the false positive animals are being considered in the calculations, once the not-infected animals (Sd, Ld e Rd) are being euthanized in a rate μ_e_, which includes 1-E (where E is the specificity of the tests).

**Vector**

**The vector insects are born susceptible (V_1_) at a rate of** Ω_f_ **and become infected upon biting infective individuals** (A_h1_, A_h2_, D_h_, A_d1_, A_d2_, A_d3_ and D_d_) **at a rate that depends on the fraction of insects that acquires infection after a bite (c_hl_, c_he_, c_cl_ and c_ce_, respectively) and the average number of bites on human (a_h_) or dog (a_c_) hosts per day (equations 22 and 23). These parameters differ because they vary in each population. The infected insects (V_2_) become infective (V_3_) at a rate of** τ, which is defined by the extrinsic incubation period**.** The vector mortality rate is given by µ_v_, µ_f_ and µ_m_ for each of its phases (V_1_, V_2_ and V_3_, respectively) **(equations 22-25)**.

**The equations that describe the model are presented below and the values of parameters are in Table 1:**

| HUMANS |  |
| --- | --- |
| $\frac{dS_{h}}{dt}= -S_{h}{ba}_{h}m_{h}V_{3}+\mu_{h}\left( L_{h}+A_{h1}+A_{h2}+D_{h}+T_{h}+R_{h} \right)+T_{h}\alpha_{h}+R_{h}\gamma_{h}$ | (1) |
| $\frac{dL_{h}}{dt}=S_{h}{ba}_{h}m_{h}V_{3}-L_{h}(\mu_{h}+{(\beta}_{h}\left( f_{m}+f_{d} \right)))$ | (2) |
| $\frac{dA_{h1}}{dt}=L_{h}\beta_{h}f_{m}-A_{h1}{(\mu}_{h}{+\delta}_{h})$ | (3) |
| $\frac{dA_{h2}}{dt}=L_{h}\beta_{h}f_{d}-A_{h2}{(\mu}_{h}{+\varphi}_{h})$ | (4) |
| $\frac{dD_{h}}{dt}=A_{h2}\varphi_{h}-D_{h}{(\mu}_{h}{+t}_{th})$ | (5) |
| $\frac{dT_{h}}{dt}=D_{h}t_{th}-T_{h}{(\mu}_{h}{+\sigma_{h}+\alpha}_{h})$ | (6) |
| $\frac{dR_{h}}{dt}=A_{h1}\delta_{h}+T_{h}\sigma_{h}-R_{h}{(\mu_{h}+\gamma}_{h})$ | (7) |
|  |  |
| DOGS |  |
| $\frac{dS_{d}}{dt}={[(\mu}_{e}+\mu_{c})\left( A_{d1}+A_{d2}+A_{d3}+A_{dc1}+A_{dc2}+A_{dc3}+D_{d}+D_{dc} \right)]+{[(\mu_{c}+\mu}_{s})\left( S_{dc}+R_{d}+R_{dc}+L_{d}+L_{dc} \right)]+{[(\mu_{c}+\mu}_{e}+\alpha_{c})\left( D_{d}+D_{dc} \right)]+\gamma_{c}R_{d}+(P_{1}+P_{2}+P_{3}+P_{4})\mu_{c}+ S_{cc}\psi- S_{d}(\rho+\omega+{ba}_{c}m_{c}V_{3})$ | (8) |
| $\frac{dL_{d}}{dt}=S_{c}{ba}_{c}m_{c}V_{3}-L_{d}\left( \mu_{c}+\mu_{s}+\omega+{(\beta}_{c}(f_{r}+f_{e}+f_{l} \right)))+L_{dc}\psi$ | (9) |
| $\frac{dA_{d1}}{dt}=L_{d}\beta_{c}f_{l}+A_{dc1}\psi-A_{d1}(\mu_{c}+\mu_{e}+\omega)$ | (10) |
| $\frac{dA_{d2}}{dt}=L_{d}\beta_{c}f_{r}+A_{dc2}\psi-A_{d2}(\mu_{c}+\mu_{e}+\omega+\delta_{c})$ | (11) |
| $\frac{dA_{d3}}{dt}=L_{d}\beta_{c}f_{e}+A_{dc3}\psi-A_{d3}(\mu_{c}+\mu_{e}+\omega+\varphi_{c})$ | (12) |
| $\frac{dD_{d}}{dt}=D_{dc}\psi+A_{d3}\varphi_{c}-D_{d}(\mu_{c}+\mu_{e}+\omega+\sigma_{c}+\alpha_{c})$ | (13) |
| $\frac{dR_{d}}{dt}=R_{dc}\psi+A_{d2}\delta_{c}+\sigma_{c}D_{d}-R_{d}\left( \mu_{c}+\mu_{s}+\omega+\rho+\gamma_{c} \right)$ | (14) |
|  |  |
| DOGS WITH COLLARS |  |
| $\frac{dS_{cc}}{dt}=R_{dc}\gamma_{c}+S_{d}\omega- S_{dc}(\mu_{c}+\psi+{ba}_{cc}m_{c}V_{3})$ | (15) |
| $\frac{dL_{cc}}{dt}=S_{dc}{ba}_{cc}m_{c}V_{3}-L_{dc}\left( \mu_{c}+\psi+{(\beta}_{c}(f_{r}+f_{e}+f_{l} \right)))+L_{d}\omega$ | (16) |
| $\frac{dA_{cc1}}{dt}=L_{dc}\beta_{c}f_{l}+A_{d1}\omega-A_{dc1}(\mu_{c}+\psi)$ | (17) |
| $\frac{dA_{cc2}}{dt}=L_{dc}\beta_{c}f_{r}+A_{d2}\omega-A_{dc2}(\mu_{c}+\psi+\delta_{c})$ | (18) |
| $\frac{dD_{cc}}{dt}=D_{d}\omega+A_{dc3}\varphi_{c}-D_{dc}(\mu_{c}+\psi+\sigma_{c}+\alpha_{c})$ | (19) |
| $\frac{dR_{cc}}{dt}=R_{d}\omega+A_{dc2}\delta_{c}+\sigma_{c}D_{dc}-R_{dc}\left( \mu_{c}+\psi+\gamma_{c} \right)$ | (20) |
|  | (21) |
| **VECTORS** |  |
| $\frac{dV_{1}}{dt}=\left( \mu_{r}+\mu_{m} \right)V_{3}+\mu_{f}V_{2}-V_{1}[c_{hl}a_{h}\left( A_{h1}+A_{h2} \right)+c_{he}a_{h}D_{h}+c_{cl}\left( \left( a_{c}\left( A_{d1}+A_{d2}+A_{d3} \right) \right)+a_{cc}\left( \left( 1-\kappa\right)\left( A_{dc1}+A_{dc2}+A_{dc3} \right) \right) \right)+c_{ce}\left( \left( 1-\kappa\right)a_{cc}D_{dc}+a_{c}D_{d} \right)]$ | (22) |
| $\frac{dV_{2}}{dt}=V_{1}\left[ c_{hl}a_{h}\left( A_{h1}+A_{h2} \right)+c_{he}a_{h}D_{h}+c_{cl}\left( \left( a_{c}\left( A_{d1}+A_{d2}+A_{d3} \right) \right)+a_{cc}\left( \left( 1-\kappa\right)\left( A_{dc1}+A_{dc2}+A_{dc3} \right) \right) \right)+c_{ce}\left( \left( 1-\kappa\right)a_{cc}D_{dc}+a_{c}D_{d} \right) \right]-V_{2}(\tau+\mu_{f})$ | (23) |
| $\frac{dV_{3}}{dt}=V_{2}\tau-V_{3}(\mu_{m}+\mu_{r})$ | (24) |

DOGS WITH VACCINE


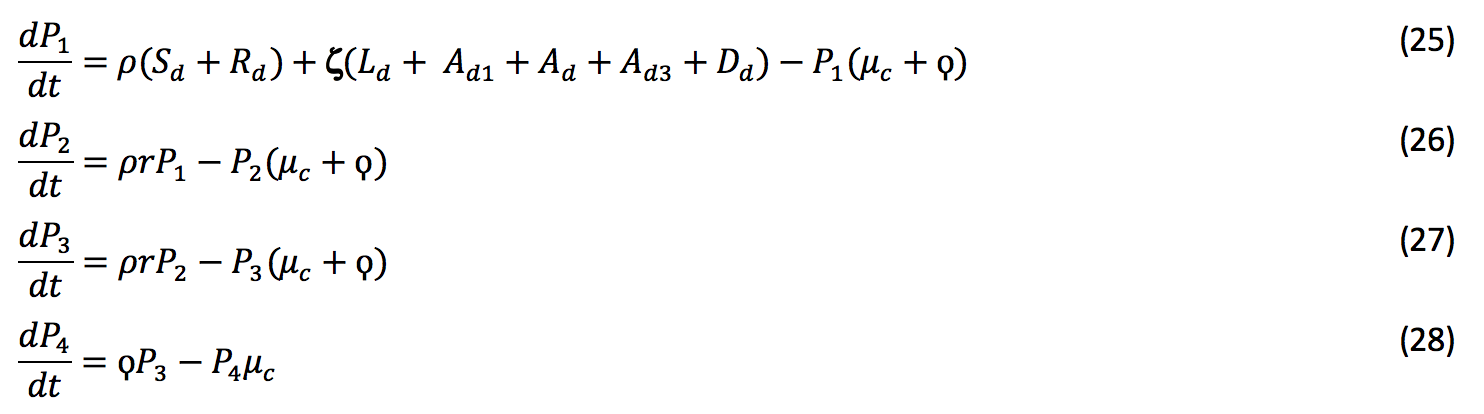


**Table 1 – Symbols and biological meanings of the parameters included in the model with the corresponding values and references**

| **SYMBOLS AND BIOLOGICAL MEANING** | | **VALUES** | **REFERENCES** |
| --- | --- | --- | --- |
| **HUMANS** | |  |  |
| µ_h_ | **Natural mortality rate** | **3.69x10^-5^ day^-1^** | **[33]** |
| α_h_ | **VL lethality** | **1.38x10^-2^ year^-1^** | [2] |
| a_h_ | **Average daily human bites by vector** | **1.4x10^-1^ day^-1^** | **[34]** |
| m_h_ | **Vector density per human** | **1.07x10^-1^** | **Assumed (based on Galvis, 2013 [34])** |
| β_h_ | **Latency period (L**A) | **3.3x10^-2^day^-1^** | **Estimated (based on Maia et al. [33])** |
| δ_h_ | **Recovery rate of asymptomatic individuals (A**R) | **9.1x10^-4^ day^-1^** | **Estimated (based on Badaro et al. [5];** Carvalho et al. [35]; Silva et al. [34]) |
| **f_m_** | **Proportion of asymptomatic individuals who recover** | **83x10^-2^** | **[38]** |
| φ_h_ | **Rate of symptom development (A**D) | **4.8x10^-3^ day^-1^** | **Estimated (based on** BRASIL, 2006) [34] |
| **f_d_** | **Proportion of individuals who become symptomatic** | **17x10^-2^** | **[38]** |
| **tt_h_** | **Treatment rate (T**R) | **2.0x10^-2^ day^-1^** | **[39]** |
| σ_h_ | **Recovery rate of treated individuals (D**R) | **1.4x10^-3^ day^-1^** | **Estimated (based on Carvalho et al. [35]; Silva et al. [34]; Alvar [38])** |
| γ_h_ | **Loss of cell-mediated immunity (R**S) | **5.47x10^-4^ day^-1^** | **Estimated (based on Badaro et al. [5];** Carvalho et al. [**35]**; Silva et al. [**34]**; Alvar **[38])** |
| Ω_h_ **Birth rate** | | α_h_ + µ_h_ |  |
| **DOGS** | |  |  |
| µ_d_ | **Natural mortality rate** | **9.23x10^-4^ day^-1^** | **[41]** |
| α_c_ | **VL lethality** | **2.12 year^-1^** | **[42]** |
| a_c_ | **Average daily dog bites by vector** | **1.4x10^-1^ day^-1^** | **[34]** |
| m_c_ | **Vector density per dog** | **1.94** | **Galvis, 2013 (oral communication)** |
| β_c_ | **Latency period (L**A) | **3.3x10^-2^ day^-1^** | **[35]** |
| f_L_ | **Proportion of individuals that remain asymptomatic** | **22x10^-2^** | **[43]** |
| δ_c_ | **Recovery rate of asymptomatic individuals (A**R) | **5.5x10^-3^ day^-1^** | **Estimated (based on** Fisa et al. [42]; Silva et al. [43]) |
| **f_R_** | **Proportion of asymptomatic individuals that recover** | **45x10^-2^** | **[43]** |
| φ_c_ | **Rate of development of symptoms (A**D) | **1.1x10^-2^ day^-1^** | **Estimated** |
| **f_e_** | **Proportion of individuals that become symptomatic** | **32x10^-2^** | **[43]** |
| σ_c_ | **Recovery rate of diseased individuals (D**R) | **2.73x10^-3^ day^-1^** | **Estimated (based on Garcia et al. [41]; Pozio et al. [40])** |
| γ_c_ | **Loss of cell-mediated immunity (R**S) | **2.73x10^-3^ day^-1^** | **Assumed** |
| Ω_c_ **Birth rate** | | α_c_ + µ_c_ |  |
| **VECTOR** | |  |  |
| µ**_v_** | **Life expectancy of non-infected vectors** | **9.09x10^-2^ day^-1^** | **[46]** |
| µ**_f_** | **Life expectancy of infected, non-infective vectors** | **1.67x10^-1^ day^-1^** | **[46]** |
| µ**_m_** | **Life expectancy of infected and infective vectors** | **2.5x10^-1^ day^-1^** | **Estimated (based on Kamhawi [47])** |
| τ | **Extrinsic incubation period** | **2.0x10^-1^ day^-1^** | **[47]** |
| **b** | **Fraction of infective bites** | **1.5x10^-1^** | **Assumed (based on Burattini et al. [31])** |
| **C_hl_** | **Proportion of insects that acquire infection after biting latent humans** | **zero** | **[48]** |
| **C_he_** | **Proportion of insects that acquire infection after biting diseased humans** | **1.2 x10^-2^** | **[48]** |
| **C_cl_** | **Proportion of insects that acquire infection after biting latent dogs** | **38.5x10^-2^** | **[49]** |
| **C_ce_** | **Proportion of insects that acquire infection after biting diseased dogs** | **24.7x10^-2^** | **[49]** |
| Ω_f_ **Birth rate** | | µ_v_ + µ_f_ + µ_m_ + µ_R_ |  |
| **SYMBOLS OF INTERVENTIONS** | |  |  |
| **µ_e_** | **Rate of really positive dog culling X S** | **Coverage X S_s_** | **[50]** |
| **µ_s_** | **Rate of false positive dog culling X (1-E)** | **Coverage X S_a_** | **[50]** |
| ρ | **Rate of really negative dogs protected by vaccination X VE X E** | **Variable** |  |
| ζ | **Rate of false negative dogs protected by vaccination X VE X (1-S)** | **Variable** |  |
| ϙ | **Rate of annual revaccination** | **365 days** |  |
| VE | **Vaccine efficacy** | **75%** | **Approximated value of vaccine efficacies of Leish-Tec^®^** [27] |
| ε | **Loss of vaccine-induced immunity** | **2.7x10^-3^ day^-1^** | **[51,52]** |
| ω | **Rate of collar use** | **Coverage** |  |
| ψ | **Loss of collar effect** | **2.8x10^-3^ day^-1^** | **[23]** |
| **a_cc_** | **Average daily collar-wearing dog bites by vector a_d_ X (1 – collar repellent effect)** | **a_d_ X 10x10^-2^** | **[23]** |
| **CE** | **Average rate of collar repellent effect** | **90x10^-2^** | **[23]** |
| **µ_R_** | **Mortality of insects that bite collar-wearing dogs** | **a_cc_ X k** |  |
| **k** | **Average collar-induced vector mortality rate** | **55x10^-2^** | **[23]** |
| **S** | **Test sensitivity in sequenced diagnostic** | **82x10^-2^** | [50] |
| **E** | **Test specificity in sequenced diagnostic** | **99x10^-2^** | [50] |
