# Supplementary material for: How much does it cost to prevent and control visceral leishmaniasis in Brazil? Comparing different measures in dogs
Source: PLoS One. 2020 Jul 21;15(7):e0236127. doi: 10.1371/journal.pone.0236127 (PMC7373293; doi:10.1371/journal.pone.0236127)
Supplement: S2 File — (DOCX) [file pone.0236127.s003.docx]

DETAILED DESCRIPTION OF THE COSTS FOR EACH MEASURE

| **INSECTICIDE-IMPREGNATED COLLAR** | | | | | | |
| --- | --- | --- | --- | --- | --- | --- |
|  |  | nº of dogs = 100 | | | semester = 1 | |
|  |  |  |  |  | |  |
|  | **material/employee** | **Quantity** | **Cost**  **(5 days)** | **Total Braz. Real** | | **Total Dolar** |
| **Labor** | Responsible veterinary | 1 | 337.5 | 337.50 | | 87.75 |
|  | Assistant employee for collaring | 2 | 230 | 460.00 | | 119.60 |
| **Material** | Scissors | 3 | 3 | 9.00 | | 2.34 |
|  | latex gloves (100 units) | 2.5 | 17 | 42.50 | | 11.05 |
|  | Insecticide-impregnated collar | 110 | 60 | 6600.00 | | 1716.00 |
| **Animal registry** | Computer | 3 | 2000 | 6000.00 | | 1560.00 |
|  | Portfolio | 104 | 3.49 | 362.96 | | 94.37 |
| **Transport** | gas fuel | 1 | 200 | 200.00 | | 52.00 |
| **TOTAL** |  |  |  | 14011.96 | | 3643.11 |

| **VACCINE** | | | | | | | | | | |
| --- | --- | --- | --- | --- | --- | --- | --- | --- | --- | --- |
|  |  | **Three first doses** | | | |  | **Revacination of 100 dogs (annual vaccination booster)** | | |  |
|  |  | **nº of evaluated dogs = 175** | | **seronegative dogs for vaccination = 100** | |  |  |  |  |  |
|  |  |  |  |  |  |  |  |  |  |  |
|  | **material/employee** | **Quantity** | **Cost**  **(1 month)** | **Total Braz. Real** | **Total Dolar** |  | **Quantity** | **Cost**  **(1 month)** | **Total Braz. Real** | **Total Dolar** |
| **Labor** | Veterinary for rapid test perform (175 dogs) | 1 | 2700 | 2700.00 | 702.00 |  | 0 | 2700 | 0.00 | 0.00 |
|  | Assistant employee for rapid test perform (175 dogs) | 2 | 1200 | 2400.00 | 624.00 |  | 0 | 1200 | 0.00 | 0.00 |
|  | Veterinary for vaccine application | 1 | 2700 | 2700.00 | 702.00 |  | 1 | 2700 | 2700.00 | 702.00 |
|  | Assistant employee for vaccine application | 1 | 2700 | 2700.00 | 702.00 |  | 1 | 2700 | 2700.00 | 702.00 |
| **Serodiagnostic material** | Disposable needle syringe | 175 | 2.65 | 463.75 | 120.58 |  | - | - | - | - |
|  | Ependorff (100 units) | 1.8 | 35 | 63.00 | 16.38 |  | - | - | - | - |
|  | Latex gloves (100 units) | 1.8 | 17 | 30.60 | 7.96 |  | - | - | - | - |
|  | Latex tube for garrote for blood collection (15m) | 1 | 16.85 | 16.85 | 4.38 |  | - | - | - | - |
|  | Vaccum tube to store blood (100 units) | 1.8 | 50.7 | 91.26 | 23.73 |  | - | - | - | - |
|  | Rapid Test (DPP®) | 175 | 7 | 1225.00 | 318.50 |  | - | - | - | - |
| **Vaccine material** | Disposable needle syringe | 300 | 3.65 | 1095.00 | 284.70 |  | 100 | 3.65 | 365.00 | 94.90 |
|  | latex gloves (100 units) | 1.2 | 17 | 20.40 | 5.30 |  |  |  |  | 0.00 |
|  | Vaccine (unit) | 306 | 90 | 27540.00 | 7160.40 |  | 102 | 90 | 9180.00 | 2386.80 |
| **Animal registry** | Computer | 3 | 2000 | 6000.00 | 1560.00 |  | 3 | 17 | 51.00 | 13.26 |
|  | Portfolio | 100 | 3.49 | 349.00 | 90.74 |  | 100 | 3.49 | 349.00 | 90.74 |
| **Transport** | gas fuel | 2 | 200 | 400.00 | 104.00 |  | 3 | 200 | 600.00 | 156.00 |
| **TOTAL** |  |  |  | 47794.86 | 12426.66 |  |  |  | 15945.00 | 4145.70 |

| **EUTHANASIA** | | | | | |
| --- | --- | --- | --- | --- | --- |
|  | **nº of evaluated dogs = 280** | | **seropositive dogs for euthanasia = 100** | | (1 year) |
|  |  |  |  |  |  |
|  | **material** | **Quantity** | **Cost (2 months)** | **Total Braz. Real** | **Total Dolar** |
| **Labor** | Veterinary for rapid test perform (280 dogs) | 1 | 2700 | 2700.00 | 702.00 |
|  | Assistant employee for rapid test perform (280 dogs) | 2 | 1200 | 2400.00 | 624.00 |
|  | Veterinary to perform euthanasia | 1 | 2700 | 2700.00 | 702.00 |
|  | Assistant employee to perform euthanasia | 1 | 2700 | 2700.00 | 702.00 |
| **Serodiagnostic material** | Disposable needle syringe | 280 | 2.65 | 742.00 | 192.92 |
|  | Disposable needle syringe | 280 | 2.65 | 742.00 | 192.92 |
|  | latex gloves (100 units) | 4.8 | 17.00 | 81.60 | 21.22 |
|  | Latex tube for garrote for blood collection (15m) | 1 | 16.85 | 16.85 | 4.38 |
|  | Vaccum tube to store blood (100 units) | 3 | 50.70 | 152.10 | 39.55 |
|  | Ependorff (100) | 3 | 35.00 | 105.00 | 27.30 |
|  | Rapid Test (DPP®) for trial | 280 | 7 | 1960.00 | 509.60 |
|  | ELISA Confirmatory test | 120 | 7 | 840.00 | 218.40 |
| **Euthanasia perform** | Latex gloves (100 units) | 1.9 | 17.00 | 32.30 | 8.40 |
|  | Cetamine 10% (50ml) * | 6.25 | 83.25 | 520.31 | 135.28 |
|  | Xilazine 2% (10ml) ** | 7.5 | 12.1 | 90.75 | 23.60 |
|  | Potassium chloride (20ml) | 100 | 0.33 | 33.00 | 8.58 |
| **Transport** | gas fuel | 3 | 200 | 600.00 | 156.00 |
| **TOTAL** |  |  |  | 16415.91 | 4268.14 |
| * 3ml/15 kgs (1 bottle for 16 dogs of 15kgs weight) | | | |  |  |
| ** 1ml/10 kgs (1 bottle for 13 dogs of 15kgs weight) | | | |  |  |
